# Cutting the molecular brakes to achieve cardiac regeneration

**DOI:** 10.1038/s41418-020-00681-z

**Published:** 2021-01-28

**Authors:** Victoria L. Nelson, Keith R. Brunt

**Affiliations:** 1 Department of Pharmacology, Dalhousie Medicine New Brunswick, Saint John, NB Canada; 2 IMPART investigator team Canada, Saint John, NB Canada

**Keywords:** Molecular biology, Biochemistry

It has been estimated that nearly 1 billion cardiomyocytes (CM) die in a typical acute myocardial infarction (MI). There is an ongoing search for safe, reliable, sufficient replacement strategy for the lost CM to restore functional mass. All organs possess some regenerative capacity but in a spectrum of ability. In some organs, this is an integral part of their continuous physiological self-renewal (e.g., blood, skin, gut). In other organs, a mixture of cell-cycle re-entry, hyperplasia, differentiation, and hypertrophy compensate for lost cell mass (e.g., liver, skeletal muscle). In the adult heart, regeneration is currently inconsequential after injury. In the absence of CM renewal, remodeling is the default (eventually leading to decompensatory heart failure). The intense proliferation of fibroblasts and scarring secure the cardiac structure but at the risk of diastolic dysfunction, dilatation, or arrhythmia on top of systolic impairment from lost contractile tissue. Fibrotic remodelling also occurs in the peri-infarct and, to a lesser degree, in remote regions of the heart. Further, the residual CM undergo concentric hypertrophy as they adapt to the increased workload leftover by lost CM, a process necessary to restore cardiac output towards normal levels. The “holy-grail” of therapy would be to survive this redistribution of hemodynamic load after injury and outrace scar formation with functional contractile capacity. A race regrettably lost far too often in the clinic.

The unrelenting hemodynamic and metabolic demands upon an adult mammalian heart don’t allow for safe cell-cycle re-entry in any functionally meaningful way—*at least naturally*. And so, facilitating any and all potential avenues to expand and bring new hope to restore lost heart muscle has been pursued earnestly [1]. There was at first burgeoning hope for undifferentiated medullary stem or progenitor cells to commit to CM. Unfortunately, this produced false flags and alas only the modification of the sterile inflammation and the paracrine milieu for neovascularization and survival of at-risk nascent CM can be certain [2, 3]. A hopeful glimmer remains for a resident progenitor cell pool in the myocardium or epicardium [4, 5]. So far, it is lacking capacity for *en masse* expansion, the ability to survive reintegration, or the engineered maturity to form with the endogenous cardiac syncytium. As the search for an autologous hero abated, new insights into induced pluripotent and transdifferentiated cells of CM lineages appeared [6, 7]. This unlimited expansion capacity from iPSC-CM or the ability for cellular conversion of non-CM into CM by transcription factor, miRNA, or epigenetic manipulation opened new opportunities. Integration challenges remain. This invariably led the field back to fundamental science for a more complete understanding and appreciation of the components—inter- and intra-cellular—necessary for heart formation. A realization took form. Better understanding of the natural course balancing hemodynamic load with metabolic demand that nature designed and refined to grow a heart to maturity could reveal the “holy-grail” approach to MI therapy. Interestingly, in some species (amphibians and fishes) [8] safe cell-cycle re-entry occurs naturally in a functionally meaningful way to elicit cardiac regeneration—so why don’t adult mammals do this?

Hauck et al. [9] sought to investigate whether CM proliferation can be regulated in a mouse model of MI through Pkm2, an important metabolic and signaling regulator in the developing heart and re-expressed after injury. This was examined by cardiac-specific Pkm2 gene knockout. Heart weight ratios and cardiomyocyte area post-MI were less than controls. Yet, there was no left ventricle dilatation nor evidence of significant fetal gene reactivation and less residual infarct scarring with an overall greater survival rate. This suggested a lack of hypertrophy induction and fibrotic remodeling with loss of CM-Pkm2, while maintaining cardiac function. Partly this was due to elevated cytoprotection afforded by improved mitochondria density and function, angiogenesis and preservation of glutathione antioxidant capacity to defend against ischemic stress post-MI. Further, loss of CM-Pkm2 prevented cytoplasmic interaction with beta-catenin (Ctnnb1) enabling nuclear transactivation of cell-cycle regulatory proteins. This interaction was verified by reciprocal co-immunoprecipitation of Pkm2 and Ctnnb1, and orthogonally verified by a Pkm2-Ctnnb1 CM-double knock-out. The net effect being that CM re-enter the cell-cycle under secure metabolic conditions to proliferate and restore functional capacity, at least in part, after MI. Consequently, this delayed fibrotic remodeling in favour of restored CM mass and function avoided much of the classical fetal gene reactivation and associated compensational concentric hypertrophy.

**Fig. 1:**
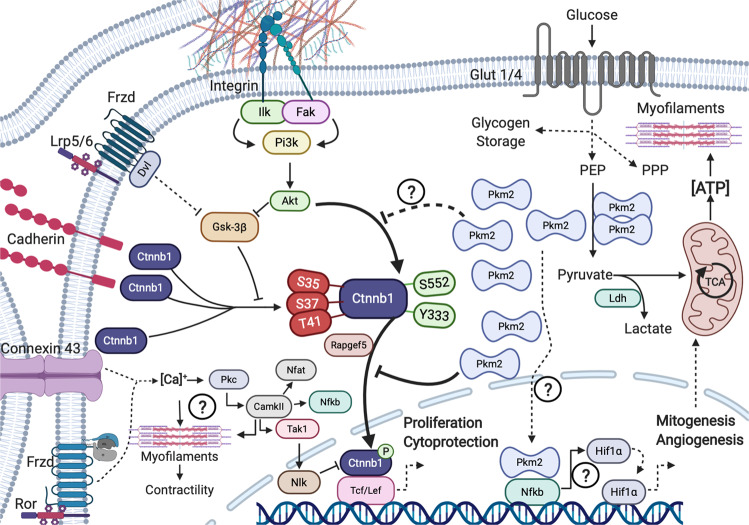
Cardiac transactivation & metabolic remodeling after MI. Diverse sources elevate cytoplasmic Ctnnb1 in cardiomyocytes after infarction and can result in isoform switching by alternative splicing to raise levels of Pkm2 (typically in dimer but also monomer/tetramer forms) resulting in restricted Ctnnb1 mediated cytoprotection and cardiomyocyte induced proliferation, with direct or secondary effects on mitogenesis, angiogenesis and ATP-bioavailability to support the energy- and macromolecule-dependent hyperplasia (Akt; protein kinase-b; CamkII: calcium-dependent protein kinase II; Ctnnb1: beta-catenin-1; Dvl: dishevelled; Fak: focal adhesion kinase; Frzd: frizzled receptor; Glut-1/4: glucose transporter-1/4; Gsk-3β: glycogen synthase kinase 3-beta; Hif1α: Hypoxia-inducible factor 1-alpha; Ilk: integrin-linked kinases; Ldh: lactate dehydrogenase; Lrp5/6: low-density lipoprotein receptor-related protein; Nfat: Nuclear factor of activated T-cells; Nfkb: Nuclear factor NF-kappa-B; Nlk: nemo-like kinase; PEP: phosphoenolpyruvate; Pi3k: phosphoinositide 3-kinase; Pkc: protein kinase C; Pkm2: muscle pyruvate kinase 2; PPP: pentose phosphate pathway; Rapgef5: rap guanine nucleotide exchange factor 5; Ror: tyrosine-protein kinase transmembrane receptor; Tak1: transforming growth factor-*β*-activated kinase 1; Tcf/Lef: T-cell factor/lymphoid enhancer-binding factor).

The molecular biology directing the switch from basal contractile CM into a non-contractile hyperplasic (fetal/neonatal) or hypertrophic (somatic) CM is complex and still not adequately understood (Fig. 1). The reason for hyperplasia to be supressed in adult CM is likely because it can either divide or contract but metabolically can’t spare the energy or withstand the mechanical forces to survive both simultaneously. As proliferation whilst contracting is unlikely, a stabilized microenvironment is essential to risk the former while foregoing the latter. Where does the balance lie between contraction and cell division in the heart in terms of force or energy? Metabolic remodelling after MI has previously been shown to involve an intrinsic ratio change by alternative splicing for Pkm1 and Pkm2. How Pkm2 accumulates in monomer, dimer, and/or tetramer form can affect its catalysis of phosphoenolpyruvate (PEP) and ADP to generate ATP. This remains unexplored in CM and non-CM of the heart and may be crucial to interpretation across studies. Indeed, in endothelial cells, tetramerization of Pkm2 or the knock-out of Pkm2 both reduce proliferation [10]. Knock-out of Pkm2 selectively in CM in the developing heart is embryonically lethal [11] due to hypoplasia with low Ctnnb1 and maladaptive hypertrophy. Magadum A et al. employed an over-expression vector to raise Pkm2 prior to an MI. Interestingly, findings similar to that of Hauck et al., shows that altering Pkm2 increases the total antioxidant capacity of cardiomyocytes and enables CM proliferation by Ctnnb1 transactivation. The outcomes of Magadum A et al. and Hauck et al. are similar but are at odds with the Pkm2 role. This may be reconciled by diverging ways limiting Pkm2 function—the former study through over accumulation of Pkm2, causing auto-inhibition by tetramer formation, and the latter study through genetic loss of function. The influence of Pkm2 on ATP-kinetics, enzymatic substrate bioavailability, influence on shifting macromolecule formation (such as the pentose phosphate pathway) are distinct from that of Pkm2 cell signaling with transcription (co)factors. The expression after MI of Pkm2 is elevated fivefold in non-CM compared to being twofold higher in CM [11]. The role of Pkm2 in CM, vascular, medullary, fibrotic cells could also be diverse. Additionally, studies have shown that Pkm2 interacts with redox-inflammatory sensitive factors, such as NF-kB and Hif1a [12] possibly by direct co-factor or synergistic functions [13] in the nucleus. With increased intracellular reactive oxygen species, Pkm2, but not Pkm1, can be inhibited by cysteine-358 oxidation [14]. Reactive oxygen species are also diverse and metabolic remodeling could produce significant redox variability in the heart affecting Pkm2 and its metabolic or cell signaling functions. There are significant roles for medullary, epicardial, fibrotic, and vascular cells in myocardial regeneration as well. Any off-target effects could influence interpretations. Other areas of interest will concern standard of care pharmacology for Pkm2. How aging, which is known to affect Ctnnb1 [15] or the regenerative potential of the heart [16] also remains to be clarified. Altering Pkm2 in other cell types could present variation in angiogenic paracrine signaling for example. It is not yet known how CM induced to proliferate are re-differentiating to resume contractile functions. Ultimately, differentiation and a return to a contractile phenotype is a critical transition in need of mechanistic exploration. Resolving comorbidity or pharmacology but also Pkm2 biochemistry with its molecular biology, in terms of temporal and cell-specificity, or even intracellular compartmentation are new avenues essential to explore. Achieving consensus on Pkm2 as a regenerative medicine target is tantalizing. Further, the work of Hauck et al. re-energizes the field of cardiac regeneration as a viable avenue for therapeutic development. For the millions facing mortality and morbidity due to MI, there is renewed hope for regenerative therapy and quality of life by such research efforts.
