# Correction to: Cutting the molecular brakes to achieve cardiac regeneration

**DOI:** 10.1038/s41418-021-00748-5

**Published:** 2021-02-19

**Authors:** Victoria L. Nelson, Keith R. Brunt

**Affiliations:** 1 Department of Pharmacology, Dalhousie Medicine New Brunswick, Saint John, NB Canada; 2 IMPART investigator team Canada, Saint John, NB Canada

**Keywords:** Molecular biology, Biochemistry

Correction to: *Cell Death & Differentiation*

10.1038/s41418-020-00681-z

The original version of this article unfortunately contained an ommission in the references that occured in production. A reference to this paper (https://www.nature.com/articles/s41418-020-00669-9) needs to be added to the start of paragraph 3, after ‘Hauck et al’. In addition, there was a typo in ‘Hauk et al’. The correct name is ‘Hauck et al’. The journal and authors apologize for the errors. The original article has been corrected.

